# Integrative Proteomics of Extracellular Vesicles from hiPSC-Derived Cardiac Organoids Reveals Heart Tissue-like Molecular Representativity

**DOI:** 10.3390/ijms27020981

**Published:** 2026-01-19

**Authors:** Carlos Miguel Vital, José Manuel Inácio, Ana Sofia Carvalho, Hans Christian Beck, Rune Matthiesen, José António Belo

**Affiliations:** 1 Stem Cells and Development Laboratory, iNOVA4Health, NOVA Medical School|Faculdade de Ciências Médicas, Universidade NOVA de Lisboa, Campo dos Mártires da Pátria, 130, 1169-056 Lisboa, Portugal; carlos.vital@nms.unl.pt (C.M.V.);; 2 Computational and Experimental Biology Laboratory, iNOVA4Health, NOVA Medical School|Faculdade de Ciências Médicas, Universidade NOVA de Lisboa, Campo dos Mártires da Pátria, 130, 1169-056 Lisboa, Portugal; 3 Department of Clinical Biochemistry and Pharmacology, Odense University Hospital, DK-5000 Odense, Denmark; hans.christian.beck@rsyd.dk

**Keywords:** extracellular vesicles, cardiac organoids, proteomics, cardiac development, disease modeling

## Abstract

Cardiovascular diseases remain a growing concern worldwide. Hence, it is critical to understand cardiac development and disease in a relevant human-based in vitro model. Human cardiac organoids are an alternative approach to studying cardiogenesis, in the context of cell–cell communication, and disease etiology, using human induced pluripotent stem cells (hiPSCs). Extracellular vesicles (EVs) are nanosized particles harboring proteins, nucleic acids, and metabolites and are implicated in intercellular communication. Since cardiac development requires a complex interplay between several cell types, we hypothesize that EVs may mediate this communication. Here, we isolated EVs from hiPSC-derived cardiac organoids (cardEVs). LC-MS/MS was performed to analyze their protein cargo and compare it with those from a cardiomyocyte cell line (AC10 CM EVs) and from human heart explants of cadaveric donors (heEVs) using a bioinformatic approach. cardEVs share 48.9% of their proteins with heEVs, with important biological processes such as “Metabolism” and “Cardiac Function” highlighted in both proteomes. This overlap between the proteomes of cardEVs and heEVs suggests a molecular similarity between the two models. Therefore, we reiterate the importance of cardiac organoids as an excellent model for studying cardiac development and disease modeling, as well as to explore the complexity of intercellular communication.

## 1. Introduction

Cardiovascular diseases (CVDs) are the leading cause of death worldwide and account for a considerable burden on socioeconomic and health systems [1]. Due to the currently aging world population, the global burden of CVD is expected to rise in the next few decades [2], underscoring the urgency for the development of new therapies. In recent decades, mouse models have been crucial for gaining an important insight into cardiovascular development [3]. However, despite the similarity between mouse and human cardiogenesis, mouse models still fall short on replicating the human cardiac system, namely, in terms of cardiac structure and physiology [4], molecular and transcriptomic regulation [5,6], and how well they modulate human disease [5,6,7]. Therefore, in vitro cardiac models have emerged as an alternative platform to study cardiovascular diseases using human cells [8]. Cardiac organoids, often called “cardioids”, are a versatile platform that allows for the integrative study of the major cardiac cell types—cardiomyocytes, endothelial cells, cardiac fibroblasts, epicardial cells—in a three-dimensional environment, closely replicating the native heart muscle structure and milieu [9,10,11]. Several iPSC-derived human cardioid models have been developed in recent years, with the goal of studying developmental events, model a specific disease, or as a platform for drug screening [12,13,14].

Given their 3D architecture and composition, organoid models are uniquely suited to study the different modes of intercellular communication. Extracellular vesicles (EVs) are nanosized particles delimited by a lipid bilayer, which have been regarded as an important mediator of intercellular communication [15,16]. These vesicles harbor metabolites, including lipids, proteins, and nucleic acids from their original cells that can be transferred to a target cell [17]. EVs can modulate the phenotype of recipient cells through receptor–ligand signaling [18], delivery of lipids to a target cell [19], gene expression modulation through transcription factor, mRNA and miRNA delivery [20,21,22], and direct protein transfer [23]. miRNAs have been the most extensively studied type of EV cargo in cardiac EV research. A study by Oh et al. investigated the miRNA expression profiles from normal and failing mouse hearts, identifying a specific subset of miRNAs that could become potential therapeutic targets in heart failure [24]. In another study, researchers explored the miRNA cargo of murine and human epicardial EVs and identified a series of miRNAs that were common to both sources, suggesting a conserved function [25]. Additionally, they also concluded that two specific miRNAs, miR-30 and miR-100, were able to elicit a similar functional improvement in injured engineered human myocardium as the EV treatment, suggesting a more potent effect of these two miRNAs [25]. Furthermore, murine cardiomyocyte-derived EVs have been reported to include an array of proteins such as heat shock proteins, involved in cardiomyocyte growth and survival [26], inflammatory factors IL-6 and TNF-α, associated with cardiac remodeling [27], and the glucose transporters GLUT1 and GLUT4 that, together with lactate dehydrogenase, are essential for carbon metabolism [28]. The cargo of cardiac EVs can also reflect disease states. For instance, EVs isolated from patients presenting with dilated cardiomyopathy were enriched in fibrinogen, serotransferrin, α-1-antitrypsin, and several apolipoproteins, when compared to healthy controls [29]. However, few studies have yet explored the proteome of cardiac EVs derived from 3-dimensional human-based platforms.

In this study, we isolated cardioid-derived EVs (cardEVs) using differential ultracentrifugation and performed a proteomic analysis of their cargo. cardEVs are enriched in proteins involved in cytoplasmic translation, energy metabolism, ECM and nucleosome organization, and are associated with highly relevant pathways such as VEGFA-VEGFR2 signaling, angiogenesis, and β1-Integrin-mediated cell surface interactions. Comparison of the cardEVs proteome with those from a human ventricular cardiomyocyte cell line (AC10 CM EVs) and from a human heart explant (heEVs) revealed a higher degree of similarity with the latter. Therefore, human cardioids offer greater physiological relevance than 2D cultures and are better suited to explore the role of EV-mediated intercellular signaling in cardiac development and disease.

## 2. Results and Discussion

### 2.1. Generation of Human Cardioids from hiPSCs

**Figure 1:**
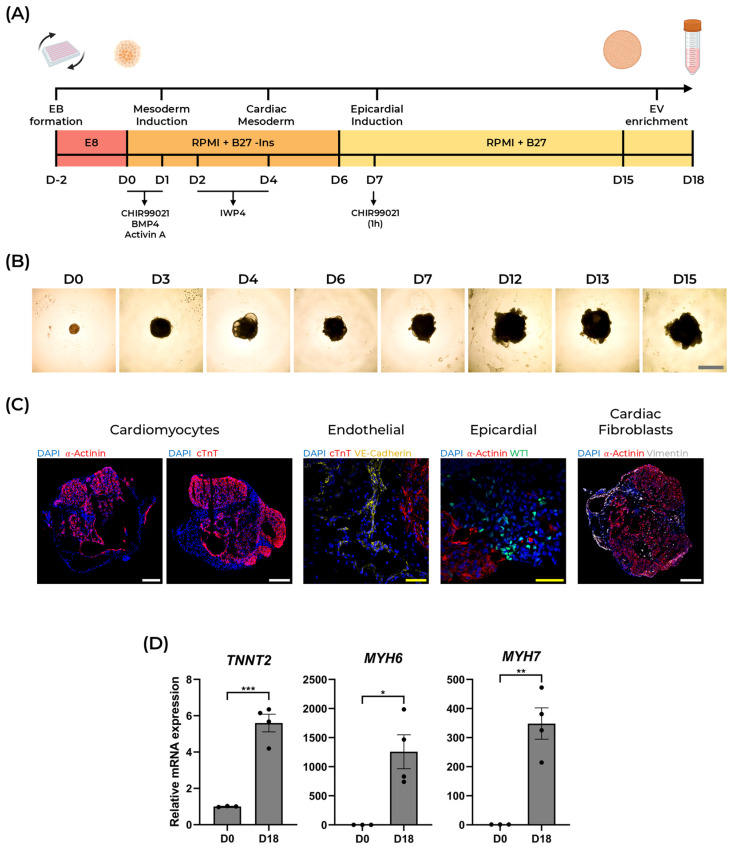
Generation of iPSC-derived human cardioids. (**A**) Schematic representation of the cardiac organoid differentiation protocol, highlighting key steps and signaling pathways modulated (Wnt, FGF, and BMP pathways). Periods of exposure to CHIR99021, BMP4, Activin A, and IWP4 are marked with black arrows. (**B**) Brightfield images of cardioids throughout differentiation. Gray scale bar = 1 mm. (**C**) Confocal micrographs of cardioid cryosections at differentiation Day 15 for α-Actinin and cTnT (red), VE-Cadherin (yellow), WT1 (green), Vimentin (gray), and DAPI (blue). *N* = 9 organoids. White scale bar = 200 μm; yellow scale bar = 50 μm. (**D**) Relative mRNA expression of key cardiac markers *TNNT2*, *MYH6*, and *MYH7*, obtained through RT-qPCR. *GAPDH* and *β-ACTIN* were used as housekeeping genes. Data presented as mean ± SEM. Unpaired t-test was performed to compare differences between D0 EBs and D18 cardioids; *, *p* < 0.05; **, *p* < 0.01; ***, *p* < 0.001. *N* = 30 organoids per replicate.

Self-organizing hiPSC-derived cardiac organoids, or human cardioids, were generated following a previously established protocol developed by Lewis-Israeli et al. [12], with some alterations. Briefly, this protocol relies on the use of small molecules and morphogens to modulate Wnt, FGF, and BMP signaling pathways to induce the differentiation of hiPSCs through mesoderm, cardiac mesoderm, and cardiac progenitor cells (Figure 1A) [12]. Embryoid bodies (EBs) were generated by centrifuging WTC cells in an ultra-low attachment 96-well plate (faCellitate, Mannhein, Germany). After 48 h, on Day 0, mesoderm induction was achieved by exposure to the Wnt pathway activator CHIR99021, as well as the morphogens BMP4 and Activin A. Cardiac mesoderm was induced by inhibiting Wnt signaling using IWP4, between Day 2 and Day 4. To promote epicardial cell differentiation, the organoids were exposed to CHIR99021 again on Day 7, concluding the 3-step Wnt pathway modulation protocol (Figure 1A).

Brightfield images demonstrated a continuous growth of the cardioids throughout differentiation (Figure 1B). Notably, cardioids started beating as early as Day 6, which was maintained until the end of the protocol (Supplementary Movie S1). Confocal imaging showed the formation of internal chambers, and the presence of α-Actinin^+^ and cTnT^+^ cardiomyocytes, VE-Cadherin^+^ endothelial cells, WT1^+^ epicardial cells, and Vimentin^+^ cardiac fibroblasts (Figure 1C). Cardiac differentiation measured by the expression of key cardiac markers *TNNT2*, *MYH6*, and *MYH7* displayed a significant upregulation at Day 18, compared to D0 (Figure 1D). This demonstrates that our protocol successfully generates functional cardiac organoids that replicate the cellular heterogeneity of heart tissue and are robustly scalable for extracellular vesicle production.

### 2.2. Isolation and Characterization of Cardioid-Derived Extracellular Vesicles (cardEVs)

Extracellular vesicles (EVs) have been regarded as an important player in intercellular communication [15]. Their cargo, mainly composed of proteins, metabolites, including lipids, and nucleic acids, can be used to modulate gene expression or alter the protein profile on a recipient cell that uptakes them [17].

**Figure 2:**
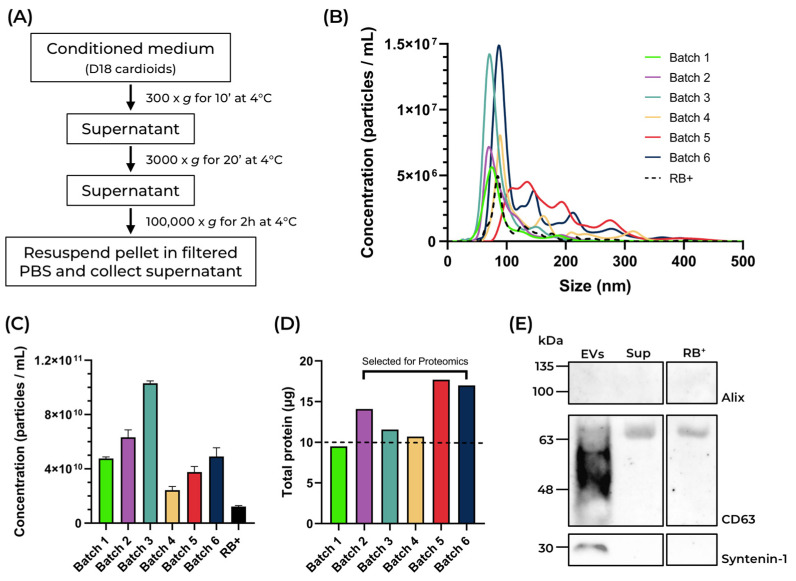
Isolation and characterization of cardEVs. (**A**) Schematic representation of the ultracentrifugation-based protocol used to isolate EVs from human cardioid conditioned medium. (**B**) Nanoparticle tracking analysis (NTA) showing the concentration and size distribution of isolated particles from the conditioned medium of human cardioids. (**C**) Particle concentration of the six differentiations of isolated particles, adjusted for the dilution factor used on the NTA. (**D**) Total protein yield for each batch of isolated particles. The batches with a protein yield ≥ 10 μg (dash line) were selected for proteomics and are indicated. (**E**) Western blot detection of the EV markers Alix, CD63, and Syntenin-1 on samples from the isolated EVs (EVs), the supernatant from ultracentrifugation (Sup), and the culture medium used to obtain the conditioned medium (RB^+^).

Here, we employed an ultracentrifugation-based protocol to isolate EVs from the conditioned medium of human cardioids, hereafter named cardEVs (Figure 2A). To obtain an EV-enriched conditioned medium, the last medium change was performed on Day 15 of differentiation, and the cardioids were cultured for an additional 3 days on the same medium (Figure 1A). On Day 18, the conditioned medium was collected and centrifuged at 300× *g* for 10 min. The supernatant was centrifuged again at 3000× *g* for 20 min. For the last step of the isolation protocol, the supernatant of the 3000× *g* centrifugation was ultracentrifuged at 100,000× *g* for 2 h, and the resulting pellet, containing the isolated particles, was resuspended in filtered PBS (Figure 2A). Nanoparticle tracking analysis (NTA) was performed to study the size and concentration of the isolated particles (Figure 2B).

We analyzed samples from six different differentiations. Overall, particle modal size varied between different batches (71.7 ± 1.2 nm to 117.7 ± 8.4 nm), with a size distribution ranging between 20 and 300 nm (Figure 2B). Particle concentrations ranged from 2.43 × 10^10^ ± 2.75 × 10^9^ to 1.03 × 10^11^ ± 1.83 × 10^9^ particles/mL (Figure 2C), with total protein yields ranging from 9.52 to 17.62 μg (Figure 2D). These results indicate that, even with slight variations in the particle concentration and protein yield, EV production and isolation remain reproducible across differentiations. We also analyzed the culture medium used at the final stages of the differentiation protocol, RB^+^. The isolated particles had a modal size of 83.1 ± 3.4 nm and a total concentration of 1.22 × 10^10^ ± 7.33 × 10^8^ particles/mL (Figure 2B). The EV identity of the isolated particles was assessed by Western blot through the detection of the classical small EV markers CD63, Alix, and Syntenin-1 [30]. In Figure 2E, we can observe strong detection of CD63, between 48 and 63 kDa, and of Syntenin-1, at 30 kDa, on the vesicles isolated from human cardioids, thereby confirming their EV identity. In contrast, neither one of these bands were detected on the supernatant from the ultracentrifugation nor the RB^+^ medium, indicating that the EV isolation protocol was effective and the culture medium did not contaminate our preparation with any CD63^+^ Syntenin-1^+^ EVs. These findings demonstrate that cardiac organoids enable the efficient production and reliable isolation of large quantities of EVs.

### 2.3. Proteomic and Functional Profiling of cardEVs

**Figure 3:**
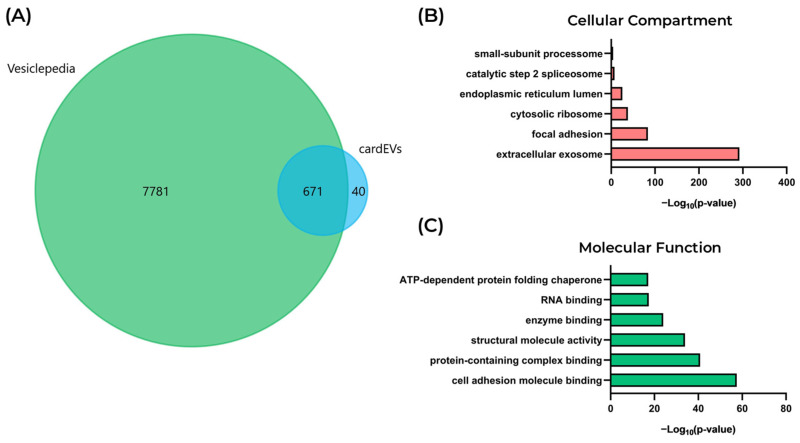
Functional profiling of the protein cargo of cardEVs. (**A**) Venn diagram comparing the cardEV proteins with EV proteins cataloged in the Vesiclepedia database. (**B**) Cellular Compartment terms enriched in cardEVs. (**C**) Molecular Function terms enriched in cardEVs.

For proteomic analysis, only EV batches yielding ≥ 10 μg of protein were considered, with five batches subsequently subjected to LC-MS/MS (Figure 2D). A total of 711 proteins were detected in cardEVs, 671 of which (94.4%) were already cataloged in the Vesiclepedia EV protein database (Figure 3A). We performed a functional analysis, where the most significantly enriched Cellular Compartment term was “extracellular exosome” (Figure 3B). Also, the most significantly enriched Molecular Function terms were “cell adhesion molecule binding”, followed by “protein-containing complex binding”, and “structural molecule activity” (Figure 3C). Altogether, these results corroborate the EV identity of the isolated particles. Next, we dissected the protein cargo of cardEVs, exploring the most abundant proteins, protein–protein interactions, and associated pathways.

In Table 1, we showcase the ten most abundant proteins in cardEVs, based on their iBAQ values. Interestingly, four out of the five most abundant proteins are histones (H2BC8, H3-3B, H4C1, and H2AC14). Histones are traditionally known for their roles in chromatin organization, as components of the nucleosome [31,32], and gene regulation, closely tied to post-translational modifications (PTMs) [32,33], both occurring within the nuclear compartment. Histones have also been identified as components of extracellular vesicles [30,34,35], although their function is still unclear. Several hypotheses have been proposed for the presence of histones in EV preparations, such as the fusion of EVs with free histones during ultracentrifugation [36], or sorting into exosomes with DNA, in a nucleosome-like configuration [37]. A recent study has demonstrated that histones H3 and H4 colocalized predominantly at the surface of a CD63^+^ population of EVs from HeLa cells [38]. Although the authors do not exclude the possibility that some of these EV histones are associated with the nucleosome, most of them are unmodified histones (lacking PTMs) and histone octamers that bind efficiently to EVs and apparently do not require DNA for this association [38]. β-Actin, ACTB, a component of the cytoskeleton [39] and major player in sarcomere structure [40], and fibronectin, FN1, a large glycoprotein involved with ECM organization [41,42], are two other proteins associated or co-isolated with EVs [30], which are abundantly present in cardEVs. Hemopexin, HPX, is a free-heme scavenger with cardioprotective effects through the mitigation of oxidative stress and inflammation [43,44], while transferrin, TF, is essential for cellular iron metabolism [45]. SLC2A3, also known as GLUT3, is an important glucose transporter crucial for cardiac development due to the high energy demands of the developing tissue [46]. Finally, midkine, MDK, is a heparin-binding growth factor expressed during mid-gestational development and in adults presenting with pathological conditions such as myocardial infarction or heart failure [47]. Besides its role during development, MDK is often associated with regeneration, modulation of ECM proteins, angiogenesis, and cardioprotection [47].

Next, we looked at how the proteins identified in cardEVs interacted with each other and what were the major pathways they were associated with. To do this, we generated a protein–protein interaction network, where proteins were grouped based on their interaction score (Figure S1). The largest protein group retrieved was “Cytoplasmic Translation”. This group included ribosomal proteins (RL14, RS13, RLA2, RS21), translation initiation factors (IF2B, EIF3D, EIF3C, EIF3A, EIF3B), and other RNA-associated proteins (Figure S1, Table S1). The “Carbon Metabolism” group predominantly consisted of enzymes involved in central metabolism (GAPDH, ALDOA, LDHA, GPI), as well as kinases (PKM, PGK1, CKB), transporters (SLC2A3, SLC16A3, SLC1A5), and proteins involved in protection against oxidative stress (PRDX1, PRDX2, PXDN) (Figure S1, Table S1). The “Extracellular Matrix Organization” group included fibronectin, FN1, as the most abundant protein in this group. In addition, collagens (COL1A2, COL2A1, COL18A1, COL6A2, COL6A3, COL6A1, COL5A1, COL4A2, COL5A2, COL4A1), other ECM components (FGA, FGB, SERPINH1, LAMC1, LAMB1, LAMA5, VCAN, NID1, FBN1), and cell surface proteins (TGFBI, IGFBP3, LGALS3, GPC3) were also heavily present (Figure S1, Table S1). The “Actin Dynamics” group included several proteins with high abundance in cardEVs, such as actin (ACTB, ACTA1, ACTG1), tubulin (TUBA1B, TUBB4B), the α-Actinin isoforms ACTN4 and ACTN1, members of the 14-3-3 protein family (YWHAZ, SFN, YWHAE, YWHAB, YWHAG, YWHAQ), and myosin (MYL6, MYL12A, MYH10) (Figure S1, Table S1). Another interesting cluster was the one from “Nucleosome Organization”, which featured several histones (H2BC4, H3-3, H4C6, H2AC14, H1-2, H1-5, H3-2, H2BC21), histone modifiers (KAT2B, NSD1), and other proteins involved in chromatin modeling (SET, HMGB2, SSRP1) (Figure S1, Table S1). Next, we explored the human biological pathways more significantly associated with the proteins identified in cardEVs (Table 2).

Notably, the three most significantly associated pathways were related to translation. This correlates well with the largest PPI groups, namely, the ones associated with “Cytoplasmic Translation” and “mRNA Splicing” (Figure S1). EVs carrying ribosomal proteins have been reported to elicit phenotypic changes in receptor cells [48,49]. We also identified several interactions between ribosomal proteins and translation initiation factors, particularly those belonging to the eukaryotic translation initiation factor 3 (eIF3) complex family (Figure S1). The eIF3 complex plays a crucial role in general translation, both in translation initiation as well as a structural scaffold, where it interacts with other eIFs, ribosomal proteins and mRNA [50]. This simultaneous presence of ribosomal and eIF proteins hints a putative translational regulation effect of cardEVs. However, the functional implications of these proteins in EVs have been reported in disease states, mostly in cancer [51,52]. Therefore, further investigation is required to elucidate the mechanistic implications of these proteins on the target cells, and more importantly, what role may they play in a physiological state.

Additionally, three other pathways were significantly implicated: “VEGFA-VEGFR2 signaling”, “Inducing angiogenesis”, and “β1-Integrin cell surface interaction”. These three pathways can be associated with angiogenesis [53,54] and, therefore, are of extremely high importance in CVD research. Microparticles derived from endothelial cells were able to induce a pro-angiogenic response mediated by a β1-integrin interaction with neighboring endothelial cells. This interaction activates the Rac1-ERK1/2-ETS1 signaling cascade, triggering the enhanced release of CCL2, a promoter of endothelial cell proliferation, migration, and angiogenesis [55,56,57]. Additionally, VEGFA/VEGFR signaling is of utmost importance in angiogenesis [54]. In endothelial cells, the binding of VEGFA to the receptor VEGFR2 leads to the activation of several pathways through downstream intermediates such as MAPK, PI3K, AKT, PlC-γ, and small GTPases [58,59]. Furthermore, several ECM components such as collagens, laminins, fibronectin, and integrins have been implicated in angiogenesis [53]. Interestingly, many of these proteins were identified in the protein–protein interaction group of “Extracellular Matrix Organization” (Figure S1, Table S1) and are also associated with the “β1-Integrin cell surface interaction” pathway (Table 2). Notably, cardEVs are enriched in proteins involved in various aspects of angiogenesis, suggesting a potential pro-angiogenic activity of cardioid-derived extracellular vesicles that may promote endothelial sprouting and stabilization of vessel-like structures within cardioids, which might reduce the formation of hypoxia-driven cores [60].

In summary, these results indicate that cardEVs are enriched in proteins involved in fundamental cellular processes, such as cytoplasmic translation, energy metabolism, ECM, and nucleosome organization. Additionally, cardEV proteins are significantly involved in pathways associated with regulation of translation and angiogenesis. Consequently, these results implicate cardEVs in the complex dynamics of cardiac physiology.

### 2.4. Comparing the Protein Cargo of cardEVs with Other Sources of Cardiac EVs

Cardiovascular research has evolved greatly in recent years regarding the study models used. The field has seen an increasing shift towards cell-based models, with the establishment of primary cardiac cell lines, and differentiation protocols for iPSC-derived culture systems. Within iPSC-derived models, the development of 3D culture systems has been evolving with the goal of replicating the most important structural and physiological features of the native heart tissue. With this in mind, we sought to understand how the proteome of our EVs, derived from 3-dimensional cardiac organoids, would compare to those of two different cardiac models: EVs isolated from the AC10 human ventricular cardiomyocyte cell line (AC10 CM EVs; [61]), and EVs isolated from human cardiac explants of cadaveric donors (heEVs; [62]).

**Figure 4:**
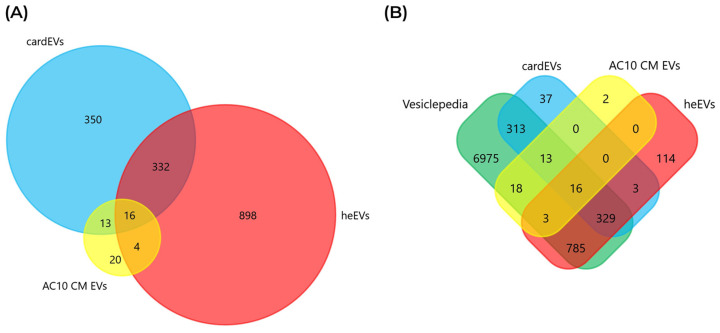
Comparison of the protein cargo of cardEVs with two other cardiac EV sources. (**A**) Venn diagram comparing the identified proteins in cardEV with EV proteins from AC10 cardiomyocytes (AC10 CM EVs) and human heart explants (heEVs). (**B**) Venn diagram comparing the cardEV, AC10 CM EV, and heEV proteins with EV proteins cataloged in the Vesiclepedia database.

Figure 4A shows a diagram comparing the number of identified proteins in each EV source, as well as the number of proteins in common between each of them. In particular, cardEVs share the most proteins, 348, with heEVs (48.9%), and 29 proteins with AC10 CM EVs (4.1%). Notably, only 16 proteins are common to the three datasets. Most of the proteins identified in the EVs isolated from AC10 CMs and heart explants were already cataloged on Vesiclepedia, with just 2 proteins from AC10 CM EVs and 117 from heEVs (3 of which were shared with cardEVs) not included in this EV protein database (Figure 4B), reinforcing their EV identity of these particles.

Interestingly, most of the 16 proteins shared between cardEVs and the other two cardiac EV sources are related to the ECM (Table S2). From those, 9 are ECM components (COL6A3, FN1, HSPG2) [42,63,64], while the others are associated with membrane transport (CLTC, VAT1) [65,66], EV biogenesis and cargo sorting (CD9, SDCBP) [67,68], metabolism (PKM, ALDH16A1) [69,70], and inflammation (S100A9) [71] (Table S2). Together, these proteins highlight how cardiac EVs integrate structural remodeling, metabolic adaptation, vesicle trafficking, and immune regulation, making them powerful mediators of cardiac homeostasis and disease [72,73,74]. From the 13 proteins exclusively shared between cardEVs and AC10 CM EVs, 11 are ECM constituents (COL6A1, COL6A2, EMILIN1, TGFBI) [75,76,77], while only APOB, a lipoprotein, and TOP1, a topoisomerase, have distinct functions [78,79] (Table S3). The four proteins exclusively shared between AC10 CM EVs and heEVs are related with membrane transport (MYOC1) [80], signaling (MYOC1, NT5E) [80,81], cell–matrix interactions (FBLN2) [82], and antigen presentation (HLA-B) [83] (Table S4). Since cardEVs and heEVs share the most proteins, and the latter are more representative of the cardiac tissue than AC10 CM EVs, we have dived further into comparing the protein cargo of cardEVs with the one from heEVs.

### 2.5. Comparison Between cardEVs and heEVs

**Figure 5:**
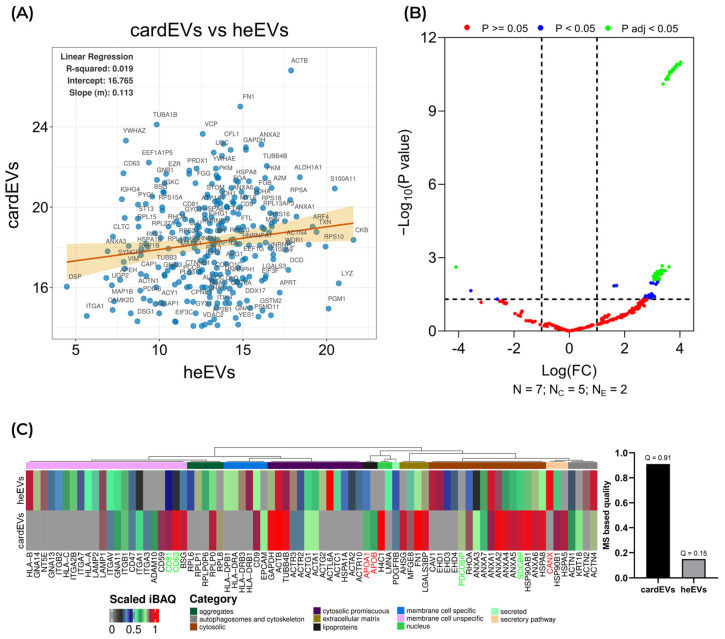
Proteomic analysis of the cargoes of cardEVs and heEVs. (**A**) Scatter plot illustrating the relationship between iBAQ values obtained from heEVs (*X*-axis) and cardEVs (*Y*-axis, Supplementary File S4) for shared proteins found in both datasets. Each point represents a unique protein, with its coordinates corresponding to its respective quantitative value in the heEVs and cardEVs datasets. A linear regression analysis was performed on these shared data points, and the regression line (orange) along with its 95% confidence interval (shaded orange area) is overlaid on the plot. The results of the linear regression are displayed in the top-left corner, indicating an R-squared value of 0.019, an intercept of 16.765, and a slope of 0.113. (**B**) Volcano plot comparing regulation of shared proteins in heEVs versus cardEVs. N: total number of samples, N_C_: total number of cardEVs replicas, N_E_: total number of heEVs samples. *p* adj: *p* value corrected for multiple testing. Total number of shared proteins compared quantitatively = 315; number of proteins with *p* < 0.05 = 83; number of proteins with *p* adj < 0.05 = 56. (**C**) Heatmap and bar plot showing EV and non-EV marker [30] expression values and quality values, respectively. The plot was generated by using version 0.0.0.9 of the R package EVqualityMS “https://github.com/ruma1974/EVqualityMS/tree/master” (accessed on 7 November 2025), R package [85]. The EV quality values Q are calculated as an average difference between EV markers and non-EV markers and scaled between 0 and 1. Specific EV markers are highlighted in green, and non-EV markers in red.

We started by assessing the abundance of the 348 proteins shared between cardEVs and heEVs [62] across both sources (Table S5). Figure 5A illustrates the relationship between iBAQ values calculated for every protein in this subset, as a measure of their absolute quantity, in cardEVs (*X*-axis) and heEVs (*Y*-axis). We performed a linear regression analysis, which produced a regression line with an R^2^ value of 0.019. This result indicates that there is a very weak positive linear correlation between the quantitative values from cardEVs and heEVs for the shared locations. As a functional implication, this suggests that although cardEVs and heEVs share a great number of proteins, they are present in different quantities across the two sources. Additionally, we analyzed the regulation of shared proteins between cardEVs and heEVs (Figure 5B). Interestingly, the proteins significantly upregulated in the heEV samples (Figure 5B, green dots, positive fold change) were found to be enriched for ‘myocardial ischemia’ markers according to DISGENET functional annotation in DAVID [84]. This finding is biologically consistent, as explant tissue inevitably experiences ischemic stress during surgical procurement and transport, stressors that are absent in the cardioid culture system. Consequently, we believe this underscores a key advantage of using cardioids. 

Then, we assessed the general quality of cardEVs and heEVs (Figure 5C). The quality assessment was performed by comparing scaled iBAQ values of known EV markers and contaminants non-EV [30] in each preparation, assigning a quality score (Q). Figure 5C shows a heatmap of all the EV markers and contaminants identified in cardEVs and heEVs, along with a bar plot showing the respective quality scores. heEVs have more EV markers; however, many of these proteins are present in lower quantities. On the other hand, cardEVs feature less EV markers, but most of them have a near maximum scaled iBAQ value and include bona fide EV markers such as CD63, CD81, Alix (PDCD6IP), and syntenin (SDCBP). This correlated well with our previous finding, reinforcing the conclusion that the abundance of shared proteins is distinct in each source. cardEVs obtained a quality score of 0.91, whereas heEVs obtained a score of 0.15. In fact, these EVs derived from cardiac explants were reported as an heterogenous population with a wide size range (30–400 nm), suggesting the presence of both exosomal and non-exosomal EVs [62]. These quality scores clearly indicate that human cardioids (cardEVs) are enriched in an EV subpopulation corresponding to sEVs, compared to heEVs.

**Figure 6:**
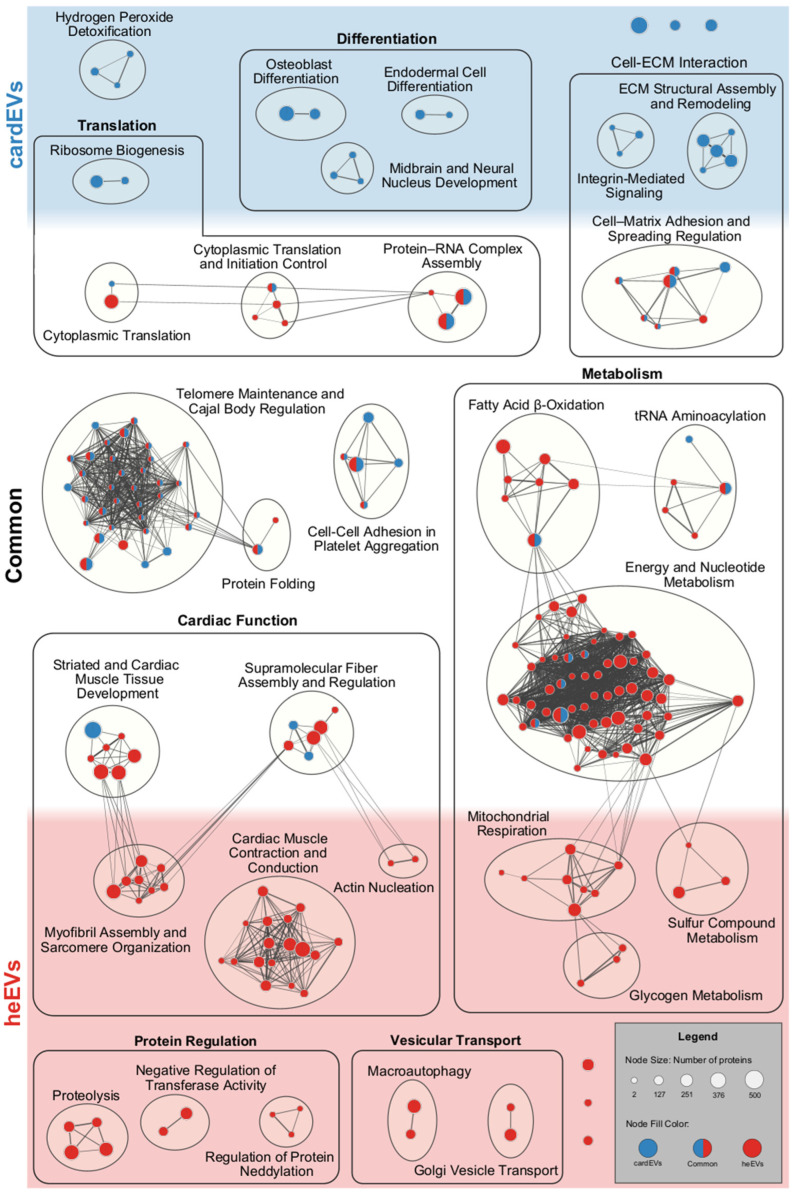
Network visualization of the biological processes identified for the protein cargo of cardEVs and heEVs. The lists of biological processes from each source were integrated in Cytoscape 3.10.4, using EnrichmentMap, to generate a single network comprising nodes associated exclusively with cardEVs (blue), heEVs (red), or common to both (red/blue). Biological process nodes were clustered together based on their coefficient of similarity and annotated using AutoAnnotate (ellipse-shaped clusters). Cluster names were generated using the Perplexity AI tool and manually adjusted. Clusters with similar functions were grouped into major biological functions (square-shaped groups).

To further explore and understand the functional implications of the protein cargo of cardEVs and heEVs, we constructed a functional enrichment network using the retrieved biological process terms for each EV source and clustered them by their similarity coefficient. Then, some clusters were grouped together into major biological processes. Figure 6 showcases the biological processes identified exclusively on cardEVs (red) or heEVs (blue), as well as those common to both datasets (red/blue).

Notably, cardEVs and heEVs share several processes associated with metabolism, cardiac function, telomere maintenance, cell–ECM, and cell–cell interactions, and translation. Additionally, cardEVs are uniquely enriched in development-related processes, suggesting a more fetal-like phenotype. On the other hand, heEVs are enriched in “Myofibril Assembly and Sarcomere Organization” and “Cardiac Muscle Contraction and Conduction” processes, functional features of more complex and mature cardiac tissues. Taking into consideration that EV proteomes are a subset of the proteomes of their cells of origin [17,86], it is understandable that these specific processes are unique to only one of the two cardiac EV sources. Being derived from iPSCs, cardiac organoids are often described as “less mature” models. In fact, the cardioids we used are comparable to age-matched human fetal cardiac tissues at the transcriptomic, structural, and cellular levels [12]. Therefore, the presence of development-associated processes in our human cardioids could be due to their less mature and developing phenotype. Since heEVs are isolated from explants of a native heart tissue, with mature and established cardiac functions and a proper cardiac structure, it is understandable that their proteome is distinct from that of cardEVs. On the other hand, it is also important to look at the biological processes in common between the two EV sources. Due to their intercellular communication role, EVs have been proposed as metabolic messengers [87]. A study performed by Garcia et al. demonstrated that upon energetic stress (glucose starvation), cardiomyocytes produced and secreted more EVs, with an increased presence of the glucose transporter GLUT4 and glycolytic enzymes, such as lactate dehydrogenase (LDH) [88,89]. Importantly, these glucose-starved cardiomyocyte-derived EVs are uptaken by endothelial cells, leading to an increase in pyruvate synthesis. Given the low energy demand of endothelial cells, pyruvate can diffuse locally to cardiomyocytes and be incorporated in their central metabolism, thereby supporting their nourishment [28]. This work establishes an important connection between two of the major cardiac cell types, where EVs are a crucial mediator. As demonstrated above, cardEVs harbor proteins implicated in cytoplasmic translation (Figure 4, Table 2). Though the functional network illustrated in Figure 6, it seems that heEVs have proteins related to this major biological function as well, suggesting a conserved role on translational regulation across two different cardiac models.

Taken together, these results demonstrate that cardEVs capture signals and biological processes characteristic of cardiac tissue-derived EVs. Therefore, cardEVs constitute a promising alternative, delivering greater physiological relevance than 2D cultures and complementing biopsy EVs in disease research.

## 3. Materials and Methods

### 3.1. Cell Culture

WTC human pluripotent stem cells (hiPSCs), deposited at the Human Pluripotent Stem Cell Registry (hPSCreg) under the name UCSFi001-A (RRID:CVCL_Y803), were cultured in Essential 8 Flex medium (Gibco, Waltham, MA, USA) on 6-well plates coated with Geltrex (Gibco, Waltham, MA, USA) and incubated at 37 °C, 5% CO_2_. Cells were split at 70–80% confluency using TrypLE Select (Gibco, Waltham, MA, USA).

### 3.2. Differentiation of 3D Self-Assembling Cadioids

The three-dimensional differentiation of hiPSCs into cardioids was performed following a previously established protocol [12], with some alterations. On Day 2, WTC cells were dissociated with Accutase (Gibco, Waltham, MA, USA), counted, and centrifuged at 200× *g* for 5 min. Cells were resuspended in Essential 8 Flex supplemented with the ROCK inhibitor RevitaCell Supplement (Gibco, Waltham, MA, USA) and seeded on round-bottom ultra-low attachment 96-well plates (faCellitate, Mannheim, Germany) at a density of 10,000 cells/well for a total volume of 100 μL/well. The plates were centrifuged at 100× *g* for 3 min to aggregate the cells and incubated at 37 °C, 5% CO_2_. On Day 1, 50 μL of culture media were carefully removed from each well (without disturbing the forming embryoid bodies), and 200 μL of Essential 8 Flex was added. On Day 0, 2/3 of the culture medium was removed and replaced with RB^−^ (RPMI Medium 1640 supplemented with B-27 Minus Insulin, Gibco, Waltham, MA, USA), containing 4 μM CHIR99021 (Stemgent, Cambridge, MA, USA), 1.25 ng/mL BMP4 (R&D Systems, Minneapolis, MN, USA), and 1 ng/mL of Activin A (Miltenyi Biotec, Bergisch Gladbach, Germany). On Day 1, the culture medium was replaced with fresh RB^−^. On Day 2, RB^−^ containing 2 μM IWP4 (Tocris Bioscience, Bristol, UK) was added, and the organoids were incubated for 48 h. On Day 4, the culture medium was renewed. On Day 6, the culture medium was replaced with RB^+^ (RPMI Medium 1640 supplemented with B-27, Gibco, Waltham, MA, USA). On Day 7, an epicardial induction step was performed by adding RB^+^ containing 2 μM CHIR99021 for 1 h. From this point on, the culture medium was renewed every 48 h until the cardioids were ready for further analysis.

### 3.3. Cardioid Sectioning and Immunofluorescence

Cardioids were harvested into a 1.5 mL tube and washed with PBS before being fixed in 4% PFA for 20 min. The organoids were washed with PBS and placed in 20% sucrose O/N. On the following day, the 20% sucrose solution was replaced with 30% sucrose solution. Once the organoids reached the bottom of the tube, they were transferred to cryomolds (Tissue-Tek, Torrance, CA, USA) and included in OCT (Avantor, Radnor, PA, USA). The organoids were cut into 12 μM sections in a Leica CM3050 S cryostat (Leica Biosystems, Nussloch, Germany).

For immunostaining, the sections were washed twice with PBS and incubated with Blocking Solution (2% BSA, 5% Donkey Serum, in PBS) for 2 h at RT. Then, they were incubated with primary antibodies (Table S6) diluted in Blocking Solution O/N at 4 °C. The sections were washed 3 times with 0.5% PBS-Triton and incubated with secondary antibodies (Table S7) diluted in Blocking Solution for 2 h at 4 °C. Next, they were washed twice with 0.5% PBS-Triton and once with PBS and incubated with DAPI diluted 1:100 in 2% BSA and 0.5% PBS-Triton for 20 min at RT. The sections were washed twice with PBS and mounted using Mowiol Mouting Medium.

Cardioid cryosection images were acquired on a Zeiss LSM710 system using a 10× 0.3 NA objective and a 40× 1.2 NA water immersion objective with ZEN Black 2011SP1 1.0.1.0. Emission windows were 490–515 nm for Alexa Fluor 488 and 580–750 nm for Alexa Fluor 568 using 488 nm and 561 nm excitation lasers, respectively.

### 3.4. RNA Isolation and RT-qPCR

Cardioids were harvested into a 1.5 mL tube, at D0 and D18, and stored in TRI Reagent (Sigma-Aldrich, St. Louis, MO, USA) at −80 °C. Total RNA extraction was performed using the Direct-Zol Miniprep Kit (Zymo Research, Irvine, CA, USA), according to the manufacturer’s instructions.

For cDNA synthesis, RevertAid Reverse Transcriptase, oligo-dT primer, RiboLock RNAse Inhibitor, and dNTPs (Thermo Fisher Scientific, Waltham, MA, USA) were used following the manufacturer’s protocol with 1000 ng of RNA. The resulting cDNA was diluted 1:10 with nuclease-free water (Ambion, Austin, TX, USA).

RT-qPCR was performed on ABI QuantStudio 5 Real-Time PCR System (Thermo Fisher Scientific, Waltham, MA, USA) using the SensiFAST SYBR Lo-ROX Kit (Meridian Bioscience, Cincinnati, OH, USA). The Cycle threshold (Ct) was determined using Design and Analysis (RUO) 3.1.0 software (Thermo Fisher App). The results were analyzed as described in Livak & Schmittgen 2001, using the 2^−ΔΔCt^ method for relative gene expression analysis [89]. The gene expression data was normalized using two housekeeping genes, *GAPDH* and *β-ACTIN*, and represented relative to a control sample (set at 1).

### 3.5. Isolation of Extracellular Vesicles by Differential Ultracentrifugation

On Day 15 of the cardioid differentiation, the culture medium was renewed for the last time by adding fresh RB^+^. The cardioids were cultured for 3 additional days, and on Day 18, the EV-enriched conditioned medium was collected. The medium was centrifuged at 300× *g* for 10 min at 4 °C to pellet remaining cells. The resulting supernatant was centrifuged at 3000× *g* for 20 min at 4 °C to pellet cells and cellular debris. The supernatant was then centrifuged at 100,000× *g* for 2 h at 4 °C to pellet the EVs. After carefully removing the supernatant, the EVs were resuspended in filtered PBS and stored in protein LoBind tubes (Eppendorf, Hamburg, Germany). EV preparations were stored at −80 °C.

### 3.6. Western Blot

Total protein from cardEVs and counterpart supernatants total protein were quantified using the Pierce BCA Protein Assay Kit (Thermo Fisher Scientific, Waltham, MA, USA). Samples were prepared with 6.30 μg and a total volume of 33.3 μL. The medium sample was prepared by diluting the same volume of supernatant used to the final volume. EV, supernatant, and medium samples were loaded onto a 10% polyacrylamide gel and separated by PAGE at a constant 15 mA for 2 h. Protein bands were transferred into a nitrocellulose membrane at 100 V for 1 h. The membrane was stained with Ponceau solution to confirm successful protein transfer and washed with 0.5% TBS-T. The membrane was cut into three sections (just above the 35 and 75 kDa marker bands) and blocked with 5% non-fat milk in TBS-T for 1 h at RT. Each membrane section was incubated O/N at 4 °C with a different primary antibody (Table S8) diluted in 5% milk in TBS-T. Membranes were incubated with secondary antibody (Table S9) diluted in TBS-T for 30 min at RT, before being imaged in a ChemiDoc Imaging System (Bio-Rad, Hercules, CA, USA) using the Amersham ECL Select Western Blotting Detection Reagent (Cytiva, Marlborough, MA, USA), according to the manufacturer’s instructions.

### 3.7. Peptide Preparation and LC-MS/MS Analysis

Frozen cardiod EV samples containing 20 µg of protein were thawed and processed using the FASP method. Lysates containing SDS and DTT were loaded onto filtering columns (Millipore, Billerica, MA, USA), washed with 8 M urea in HEPES buffer [90], alkylated with IAA, and digested overnight with sequencing-grade trypsin (Promega, Madison, WI, USA). Subsequently, 5 µL of peptides was analyzed by nano-LC-MS/MS using a Dionex RSLCnano 3000 coupled to an Exploris 480 Orbitrap mass spectrometer (Thermo Scientific), as previously described [90]. Samples were loaded onto a custom fused capillary precolumn (2 cm length, 360 µm OD, 75 µm ID; ReproSil Pur C18 5.0 µm resin, Dr. Maish, Ammerbuch-Entringen, Germany) at 5 µL/min for 6 min. Separation was performed on a custom fused capillary column (25 cm length, 360 µm OD, 75 µm ID; ReproSil Pur C18 1.9 µm resin, Dr. Maish) at 250 nL/min using a linear gradient from 89% Buffer A (0.1% formic acid) to 32% Buffer B (0.1% formic acid in 80% acetonitrile) over 56 min. Mass spectra were acquired in positive ion mode with a 2 s cycle time, switching between an Orbitrap survey scan (350–1200 *m*/*z*) and HCD fragmentation (NCE 30%) in the ion routing multipole. Maximum injection times were set to “Auto,” with an ion selection threshold of 10,000 counts and dynamic exclusion of 30 s.

### 3.8. Analysis of Proteomics Data

Ten LC-MS runs of cardiod EVs were processed using VEMS [91] and MaxQuant 2.1.0.0 [92], with a 1% false discovery rate (FDR) for peptide and protein identification. Variable modifications included methionine oxidation, lysine acetylation, and N-terminal protein acetylation, allowing for up to four missed trypsin cleavages. VEMS settings specified a 5 ppm mass accuracy for precursor ions and 0.01 *m*/*z* for fragment ions, while all other MaxQuant v.2.1.0.0 settings remained at default values. For quantitative analysis, intensity-based absolute quantification (iBAQ) was calculated by dividing total ion counts by the number of theoretical canonical tryptic peptides (excluding missed cleavages). Quantitative data from VEMS was analyzed using the R statistical programming language. Preprocessing involved log_2_(x + 1) transformation and quantile normalization of protein label-free expression values. Finally, sEVs were characterized, and EV and non-EV markers [30] were characterized using the EVqualityMS tool (https://github.com/ruma1974/EVqualityMS/tree/master (accessed on 10 November 2025) [85].

### 3.9. Functional and Protein Interaction Analyses of the EV Cargoes of cardEVs and heEVs

The protein lists for cardEVs, AC10 CM EVs [61], and heEVs [62] were manually curated by retrieving the UniProt ID, gene name, and ENSG code for every entry using ID Mapping (https://www.uniprot.org/id-mapping (accessed on 10 November 2025)), BioMart (https://www.ensembl.org/biomart/martview/fbac87c392f98ce9c02428324deb31a1 (accessed on 10 November 2025)), and g:Convert (https://biit.cs.ut.ee/gprofiler/convert (accessed on 10 November 2025)) tools.

Functional analysis for Gene Ontology biological process (BP), Molecular Function (MF), and Cellular Compartment (CC) was performed using the g:Profiler tool (https://biit.cs.ut.ee/gprofiler/gost (accessed on 11 November 2025)). Network imaging of the biological processes identified in cardEVs and heEVs were visualized in Cytoscape 3.10.4 [93].

To generate a protein–protein interaction (PPI) network, a list of the proteins identified in cardEVs was uploaded to the STRING web tool (https://string-db.org (accessed on 12 December 2025)) and visualized in Cytoscape 3.10.4. Next, we imported a human protein interactome (HumanNet v3–FN) from the NDEx WebApp (https://www.ndexbio.org/ (accessed on 12 December 2025)) and merged it with our own PPI network to include other curated human protein interactions, besides the ones retrieved from the STRING app. Proteins were grouped based on their interaction scores and named using AutoAnnotate, with the “Label column” parameter set to “stringdb::primary description”.

### 3.10. Statistics

Statistical analyses were performed using the R statistical programming language. Label-free quantitative (LFQ) values for heEVs were retrieved from [62] and converted to iBAQ as described in Section 3.8 (Supplementary File S4). The data subsequently underwent log_2_(x + 1) transformation and quantile normalization. To compare iBAQ expression values between cardEVs and heEVs, simple linear regression was applied using a linear regression model implemented in base R. To analyze the regulation of shared protein between cardEVs and heEVs, a pairwise comparison was performed using the limma R package following log2 transformation and sum normalization (Supplementary File S5).

## 4. Conclusions

This study demonstrates that human cardiac organoids provide a valuable model for studying intercellular communication during cardiac development, as their secreted EVs (cardEVs) exhibit proteomic similarities to those derived from human heart explants.

We successfully generated cardiac organoids from hiPSCs, which were composed of the most important cardiac cell types (cardiomyocytes, endothelial cells, epicardial cells, cardiac fibroblasts) and displayed important functional features of the ventricular myocardium. The proteome of EVs produced by these organoids was analyzed and compared to two other EV proteomes from other cardiac sources: a cardiomyocyte cell line and human heart explants. cardEVs and heEVs shared a significant overlap in protein composition. cardEVs and heEVs shared proteins involved in metabolism, translation, and, most importantly, cardiac function. At the same time, cardEVs were enriched in cell–ECM interaction and early development-associated proteins.

These findings establish human cardiac organoids as a promising platform for investigating EV-mediated mechanisms underlying both cardiac development and disease.

## Figures and Tables

**Table 1:** Ten most abundant proteins identified in cardEVs, according to their iBAQ values.

Gene Name	iBAQ	Function	Ref.	Present in Vesiclepedia
*H2BC8*	181,341,427.50	Component of the nucleosome	[31]	Yes
*H3-3B*	136,707,625.00	Component of the nucleosome	[31]	Yes
*H4C1*	126,990,328.75	Component of the nucleosome	[31]	Yes
*ACTB*	118,332,848.75	Contributes to sarcomere formation and contractile function	[39,40]	Yes
*H2AC14*	61,760,312.50	Component of the nucleosome	[31]	Yes
*FN1*	34,271,842.50	ECM glycoprotein; mediates cell–matrix adhesion	[41,42]	Yes
*HPX*	24,905,483.63	Heme-binding protein	[43,44]	Yes
*TF*	23,317,167.38	Iron-binding transport protein	[45]	Yes
*SLC2A3*	22,775,432.50	Glucose transporter 3 (GLUT3)	[46]	Yes
*MDK*	22,014,288.75	Heparin-binding growth factor	[47]	Yes

**Table 2:** Most relevant pathways associated with the protein identified in cardEVs.

Pathway	Source	Unique Genes	Similarity	*p*-Value
Translation elongation and termination	Signor	61/132	0.22	1.19 × 10^−37^
Initiation of Translation	Signor	55/113	0.21	1.47 × 10^−35^
Cytoplasmic ribosomal proteins	WikiPathways	47/88	0.21	9.84 × 10^−33^
VEGFA-VEGFR2 signaling	WikiPathways	86/436	0.15	1.92 × 10^−22^
Inducing angiogenesis	WikiPathways	87/487	0.14	9.19 × 10^−20^
β1-Integrin cell surface interaction	NCI PID	31/65	0.13	1.65 × 10^−19^

## Data Availability

The original data presented in the study is openly available in ProteomeXchange Consortium [94] via the PRIDE [95] partner repository at 10.6019/PXD072207 or PXD072207.

## Supplementary Materials

The following supporting information can be downloaded at https://www.mdpi.com/article/10.3390/ijms27020981/s1.

## Abbreviations

The following abbreviations are used in this manuscript:

hiPSCs	Human induced pluripotent stem cells
EVs	Extracellular vesicles
LC-MS/MS	Liquid-chromatography tandem mass spectrometry
CVD	Cardiovascular diseases
cardEVs	EVs isolated from human cardioids
heEVs	EVs isolated from human heart explants of cadaveric donors
AC10 CM EVs	EVs isolated from the AC10 cardiomyocyte cell line
PTM	Post-translational modification
ECM	Extracellular matrix
iBAQ	Intensity-based absolute quantification

## References

[1] Roth G.A., Mensah G.A., Johnson C.O., Addolorato G., Ammirati E., Baddour L.M., Barengo N.C., Beaton A.Z., Benjamin E.J., Benziger C.P. (2020). Global Burden of Cardiovascular Diseases and Risk Factors, 1990–2019: Update from the GBD 2019 Study. J. Am. Coll. Cardiol..

[2] Chong B., Jayabaskaran J., Jauhari S.M., Chan S.P., Goh R., Kueh M.T.W., Li H., Chin Y.H., Kong G., Anand V.V. (2025). Global Burden of Cardiovascular Diseases: Projections from 2025 to 2050. Eur. J. Prev. Cardiol..

[3] Krishnan A., Samtani R., Dhanantwari P., Lee E., Yamada S., Shiota K., Donofrio M.T., Leatherbury L., Lo C.W. (2014). A Detailed Comparison of Mouse and Human Cardiac Development. Pediatr. Res..

[4] Oh J.G., Kho C., Hajjar R.J., Ishikawa K. (2019). Experimental Models of Cardiac Physiology and Pathology. Heart Fail. Rev..

[5] Vakrou S., Liu Y., Zhu L., Greenland G.V., Simsek B., Hebl V.B., Guan Y., Woldemichael K., Talbot C.C., Aon M.A. (2021). Differences in Molecular Phenotype in Mouse and Human Hypertrophic Cardiomyopathy. Sci. Rep..

[6] von Scheidt M., Zhao Y., Kurt Z., Pan C., Zeng L., Yang X., Schunkert H., Lusis A.J. (2017). Applications and Limitations of Mouse Models for Understanding Human Atherosclerosis. Cell Metab..

[7] Shin H.S., Shin H.H., Shudo Y. (2021). Current Status and Limitations of Myocardial Infarction Large Animal Models in Cardiovascular Translational Research. Front. Bioeng. Biotechnol..

[8] Karakikes I., Ameen M., Termglinchan V., Wu J.C. (2015). Human Induced Pluripotent Stem Cell-Derived Cardiomyocytes: Insights into Molecular, Cellular, and Functional Phenotypes. Circ. Res..

[9] Yang J., Lei W., Xiao Y., Tan S., Yang J., Lin Y., Yang Z., Zhao D., Zhang C., Shen Z. (2024). Generation of Human Vascularized and Chambered Cardiac Organoids for Cardiac Disease Modelling and Drug Evaluation. Cell Prolif..

[10] Branco M.A., Dias T.P., Cabral J.M.S., Pinto-do-Ó P., Diogo M.M. (2022). Human Multilineage Pro-Epicardium/Foregut Organoids Support the Development of an Epicardium/Myocardium Organoid. Nat. Commun..

[11] Meier A.B., Zawada D., De Angelis M.T., Martens L.D., Santamaria G., Zengerle S., Nowak-Imialek M., Kornherr J., Zhang F., Tian Q. (2023). Epicardioid Single-Cell Genomics Uncovers Principles of Human Epicardium Biology in Heart Development and Disease. Nat. Biotechnol..

[12] Lewis-Israeli Y.R., Wasserman A.H., Gabalski M.A., Volmert B.D., Ming Y., Ball K.A., Yang W., Zou J., Ni G., Pajares N. (2021). Self-Assembling Human Heart Organoids for the Modeling of Cardiac Development and Congenital Heart Disease. Nat. Commun..

[13] Zhu Y., Yang S., Zhang T., Ge Y., Wan X., Liang G. (2024). Cardiac Organoids: A 3D Technology for Disease Modeling and Drug Screening. Curr. Med. Chem..

[14] Li T., Yin J., Hao Y., Gao W., Li Q., Feng Q., Tao B., Hao M., Liu Y., Lin C. (2025). Single-Cell Sequencing and Organoids: Applications in Organ Development and Disease. Mol. Biomed..

[15] Van Niel G., D’Angelo G., Raposo G. (2018). Shedding Light on the Cell Biology of Extracellular Vesicles. Nat. Rev. Mol. Cell Biol..

[16] Maacha S., Bhat A.A., Jimenez L., Raza A., Haris M., Uddin S., Grivel J.C. (2019). Extracellular Vesicles-Mediated Intercellular Communication: Roles in the Tumor Microenvironment and Anti-Cancer Drug Resistance. Mol. Cancer.

[17] Dixson A.C., Dawson T.R., Di Vizio D., Weaver A.M. (2023). Context-Specific Regulation of Extracellular Vesicle Biogenesis and Cargo Selection. Nat. Rev. Mol. Cell Biol..

[18] Jahnke K., Staufer O. (2024). Membranes on the Move: The Functional Role of the Extracellular Vesicle Membrane for Contact-Dependent Cellular Signalling. J. Extracell. Vesicles.

[19] Ghadami S., Dellinger K. (2023). The Lipid Composition of Extracellular Vesicles: Applications in Diagnostics and Therapeutic Delivery. Front. Mol. Biosci..

[20] Pando A., Schorl C., Fast L.D., Reagan J.L. (2023). Tumor Derived Extracellular Vesicles Modulate Gene Expression in T Cells. Gene.

[21] Tian C., Gao L., Rudebush T.L., Yu L., Zucker I.H. (2022). Extracellular Vesicles Regulate Sympatho-Excitation by Nrf2 in Heart Failure. Circ. Res..

[22] Tian C., Hu G., Gao L., Hackfort B.T., Zucker I.H. (2020). Extracellular Vesicular MicroRNA-27a* Contributes to Cardiac Hypertrophy in Chronic Heart Failure. J. Mol. Cell. Cardiol..

[23] Fujioka T., Nishimura T., Kawana H., Hirosawa K.M., Yamakawa R., Sapili H., Oono-Yakura K., Nakagawa R., Inoue T., Nureki O. (2025). Efficient Cellular Transformation via Protein Delivery through the Protrusion-Derived Extracellular Vesicles. Nat. Commun..

[24] Oh J.G., Lee P., Gordon R.E., Sahoo S., Kho C., Jeong D. (2020). Analysis of Extracellular Vesicle MiRNA Profiles in Heart Failure. J. Cell. Mol. Med..

[25] Del Campo C.V., Liaw N.Y., Gunadasa-Rohling M., Matthaei M., Braga L., Kennedy T., Salinas G., Voigt N., Giacca M., Zimmermann W.H. (2022). Regenerative Potential of Epicardium-Derived Extracellular Vesicles Mediated by Conserved MiRNA Transfer. Cardiovasc. Res..

[26] Gupta S., Knowlton A.A. (2007). HSP60 Trafficking in Adult Cardiac Myocytes: Role of the Exosomal Pathway. Am. J. Physiol.-Heart Circ. Physiol..

[27] Yu X., Deng L., Wang D., Li N., Chen X., Cheng X., Yuan J., Gao X., Liao M., Wang M. (2012). Mechanism of TNF-α Autocrine Effects in Hypoxic Cardiomyocytes: Initiated by Hypoxia Inducible Factor 1α, Presented by Exosomes. J. Mol. Cell. Cardiol..

[28] Garcia N.A., Moncayo-Arlandi J., Sepulveda P., Diez-Juan A. (2016). Cardiomyocyte Exosomes Regulate Glycolytic Flux in Endothelium by Direct Transfer of GLUT Transporters and Glycolytic Enzymes. Cardiovasc. Res..

[29] Roura S., Gámez-Valero A., Lupón J., Gálvez-Montón C., Borràs F.E., Bayes-Genis A. (2018). Proteomic Signature of Circulating Extracellular Vesicles in Dilated Cardiomyopathy. Lab. Investig..

[30] Welsh J.A., Goberdhan D.C.I., O’Driscoll L., Buzas E.I., Blenkiron C., Bussolati B., Cai H., Di Vizio D., Driedonks T.A.P., Erdbrügger U. (2024). Minimal Information for Studies of Extracellular Vesicles (MISEV2023): From Basic to Advanced Approaches. J. Extracell. Vesicles.

[31] Kornberg R.D. (1974). Chromatin Structure: A Repeating Unit of Histones and DNA. Science.

[32] Voss A.J., Korb E. (2025). The ABCs of the H2Bs: The Histone H2B Sequences, Variants, and Modifications. Trends Genet..

[33] Millán-Zambrano G., Burton A., Bannister A.J., Schneider R. (2022). Histone Post-Translational Modifications—Cause and Consequence of Genome Function. Nat. Rev. Genet..

[34] Ghanam J., Chetty V.K., Anchan S., Reetz L., Yang Q., Rideau E., Liu X., Lieberwirth I., Wrobeln A., Hoyer P. (2023). Extracellular Vesicles Transfer Chromatin-like Structures That Induce Non-Mutational Dysfunction of P53 in Bone Marrow Stem Cells. Cell Discov..

[35] Lázaro-Ibáñez E., Lässer C., Shelke G.V., Crescitelli R., Jang S.C., Cvjetkovic A., García-Rodríguez A., Lötvall J. (2019). DNA Analysis of Low- and High-Density Fractions Defines Heterogeneous Subpopulations of Small Extracellular Vesicles Based on Their DNA Cargo and Topology. J. Extracell. Vesicles.

[36] Jeppesen D.K., Fenix A.M., Franklin J.L., Higginbotham J.N., Zhang Q., Zimmerman L.J., Liebler D.C., Ping J., Liu Q., Evans R. (2019). Reassessment of Exosome Composition. Cell.

[37] Colombo M., Raposo G., Théry C. (2014). Biogenesis, Secretion, and Intercellular Interactions of Exosomes and Other Extracellular Vesicles. Annu. Rev. Cell Dev. Biol..

[38] Singh B., Fredriksson Sundbom M., Muthukrishnan U., Natarajan B., Stransky S., Görgens A., Nordin J.Z., Wiklander O.P.B., Sandblad L., Sidoli S. (2025). Extracellular Histones as Exosome Membrane Proteins Regulated by Cell Stress. J. Extracell. Vesicles.

[39] Small J.V. (1988). The Actin Cytoskeleton. Electron. Microsc. Rev..

[40] Skwarek-Maruszewska A., Hotulainen P., Mattila P.K., Lappalainen P. (2009). Contractility-Dependent Actin Dynamics in Cardiomyocyte Sarcomeres. J. Cell Sci..

[41] Proctor R.A. (1987). Fibronectin: A Brief Overview of Its Structure, Function, and Physiology. Rev. Infect. Dis..

[42] Zollinger A.J., Smith M.L. (2017). Fibronectin, the Extracellular Glue. Matrix Biol..

[43] Ingoglia G., Sag C.M., Rex N., De Franceschi L., Vinchi F., Cimino J., Petrillo S., Wagner S., Kreitmeier K., Silengo L. (2017). Hemopexin Counteracts Systolic Dysfunction Induced by Heme-Driven Oxidative Stress. Free Radic. Biol. Med..

[44] Li Y., Chen R., Wang C., Deng J., Luo S. (2023). Double-Edged Functions of Hemopexin in Hematological Related Diseases: From Basic Mechanisms to Clinical Application. Front. Immunol..

[45] Lakhal-Littleton S. (2019). Mechanisms of Cardiac Iron Homeostasis and Their Importance to Heart Function. Free Radic. Biol. Med..

[46] Szablewski L. (2017). Glucose Transporters in Healthy Heart and in Cardiac Disease. Int. J. Cardiol..

[47] Woulfe K.C., Sucharov C.C. (2017). Midkine’s Role in Cardiac Pathology. J. Cardiovasc. Dev. Dis..

[48] Graifer D., Malygin A., Shefer A., Tamkovich S. (2025). Ribosomal Proteins as Exosomal Cargo: Random Passengers or Crucial Players in Carcinogenesis?. Adv. Biol..

[49] Ochkasova A., Arbuzov G., Malygin A., Graifer D. (2023). Two “Edges” in Our Knowledge on the Functions of Ribosomal Proteins: The Revealed Contributions of Their Regions to Translation Mechanisms and the Issues of Their Extracellular Transport by Exosomes. Int. J. Mol. Sci..

[50] Valášek L.S., Zeman J., Wagner S., Beznosková P., Pavlíková Z., Mohammad M.P., Hronová V., Herrmannová A., Hashem Y., Gunišová S. (2017). Embraced by EIF3: Structural and Functional Insights into the Roles of EIF3 across the Translation Cycle. Nucleic Acids Res..

[51] Tey S.K., Wong S.W.K., Yeung C.L.S., Li J.Y.K., Mao X., Chung C.Y.S., Yam J.W.P. (2022). Liver Cancer Cells with Nuclear MET Overexpression Release Translation Regulatory Protein-Enriched Extracellular Vesicles Exhibit Metastasis Promoting Activity. J. Extracell. Biol..

[52] Dabbah M., Lishner M., Jarchowsky-Dolberg O., Tartakover-Matalon S., Brin Y.S., Pasmanik-Chor M., Neumann A., Drucker L. (2021). Ribosomal Proteins as Distinct “Passengers” of Microvesicles: New Semantics in Myeloma and Mesenchymal Stem Cells’ Communication. Transl. Res..

[53] Neve A., Cantatore F.P., Maruotti N., Corrado A., Ribatti D. (2014). Extracellular Matrix Modulates Angiogenesis in Physiological and Pathological Conditions. BioMed Res. Int..

[54] Abhinand C.S., Raju R., Soumya S.J., Arya P.S., Sudhakaran P.R. (2016). VEGF-A/VEGFR2 Signaling Network in Endothelial Cells Relevant to Angiogenesis. J. Cell Commun. Signal..

[55] Arderiu G., Peña E., Badimon L. (2015). Angiogenic Microvascular Endothelial Cells Release Microparticles Rich in Tissue Factor That Promotes Postischemic Collateral Vessel Formation. Arter. Thromb. Vasc. Biol..

[56] Peng Z., Pang H., Wu H., Peng X., Tan Q., Lin S., Wei B. (2023). CCL2 Promotes Proliferation, Migration and Angiogenesis through the MAPK/ERK1/2/MMP9, PI3K/AKT, Wnt/Β-catenin Signaling Pathways in HUVECs. Exp. Ther. Med..

[57] Ateeq M., Broadwin M., Sellke F.W., Abid M.R. (2024). Extracellular Vesicles’ Role in Angiogenesis and Altering Angiogenic Signaling. Med. Sci..

[58] Lohela M., Bry M., Tammela T., Alitalo K. (2009). VEGFs and Receptors Involved in Angiogenesis versus Lymphangiogenesis. Curr. Opin. Cell Biol..

[59] Shibuya M., Claesson-Welsh L. (2006). Signal Transduction by VEGF Receptors in Regulation of Angiogenesis and Lymphangiogenesis. Exp. Cell Res..

[60] Wörsdörfer P., Ergün S. (2021). The Impact of Oxygen Availability and Multilineage Communication on Organoid Maturation. Antioxid. Redox Signal..

[61] Ontoria-Oviedo I., Dorronsoro A., Sánchez R., Ciria M., Gómez-Ferrer M., Buigues M., Grueso E., Tejedor S., García-García F., González-King H. (2018). Extracellular Vesicles Secreted by Hypoxic AC10 Cardiomyocytes Modulate Fibroblast Cell Motility. Front. Cardiovasc. Med..

[62] Leitolis A., Suss P.H., Roderjan J.G., Angulski A.B.B., da Costa F.D.A., Stimamiglio M.A., Correa A. (2019). Human Heart Explant-Derived Extracellular Vesicles: Characterization and Effects on the In Vitro Recellularization of Decellularized Heart Valves. Int. J. Mol. Sci..

[63] Wang J., Pan W. (2020). The Biological Role of the Collagen Alpha-3 (VI) Chain and Its Cleaved C5 Domain Fragment Endotrophin in Cancer. OncoTargets Ther..

[64] Johnson B.B., Cosson M.V., Tsansizi L.I., Holmes T.L., Gilmore T., Hampton K., Song O.R., Vo N.T.N., Nasir A., Chabronova A. (2024). Perlecan (HSPG2) Promotes Structural, Contractile, and Metabolic Development of Human Cardiomyocytes. Cell Rep..

[65] Kaksonen M., Roux A. (2018). Mechanisms of Clathrin-Mediated Endocytosis. Nat. Rev. Mol. Cell Biol..

[66] Linial M., Miller K., Scheller R.H. (1989). VAT 1: An Abundant Membrane Protein from Torpedo Cholinergic Synaptic Vesicles. Neuron.

[67] Suárez H., Andreu Z., Mazzeo C., Toribio V., Pérez-Rivera A.E., López-Martín S., García-Silva S., Hurtado B., Morato E., Peláez L. (2021). CD9 Inhibition Reveals a Functional Connection of Extracellular Vesicle Secretion with Mitophagy in Melanoma Cells. J. Extracell. Vesicles.

[68] Lee K.M., Seo E.C., Lee J.H., Kim H.J., Hwangbo C. (2023). The Multifunctional Protein Syntenin-1: Regulator of Exosome Biogenesis, Cellular Function, and Tumor Progression. Int. J. Mol. Sci..

[69] Xiong Y., Lei Q.Y., Zhao S., Guan K.L. (2011). Regulation of Glycolysis and Gluconeogenesis by Acetylation of PKM and PEPCK. Cold Spring Harb. Symp. Quant. Biol..

[70] Charkoftaki G., Chen Y., Han M., Sandoval M., Yu X., Zhao H., Orlicky D.J., Thompson D.C., Vasiliou V. (2017). Transcriptomic Analysis and Plasma Metabolomics in Aldh16a1-Null Mice Reveals a Potential Role of ALDH16A1 in Renal Function. Chem.-Biol. Interact..

[71] Gebhardt C., Németh J., Angel P., Hess J. (2006). S100A8 and S100A9 in Inflammation and Cancer. Biochem. Pharmacol..

[72] Al Halawani A., Mithieux S.M., Yeo G.C., Hosseini-Beheshti E., Weiss A.S. (2022). Extracellular Vesicles: Interplay with the Extracellular Matrix and Modulated Cell Responses. Int. J. Mol. Sci..

[73] Xiao J., Sluijter J.P.G. (2025). Extracellular Vesicles in Cardiovascular Homeostasis and Disease: Potential Role in Diagnosis and Therapy. Nat. Rev. Cardiol..

[74] Rudsari H.K., O’Hern C., Ural E., Damrath M., Neeb E., Zoofaghari M., Veletić M., Louch W.E., Aguirre A., Balasingham I. Human Heart Organoid-Derived Extracellular Vesicles for Cardiac Intercellular Communication. Proceedings of the 10th ACM International Conference on Nanoscale Computing and Communication.

[75] Fitzgerald J., Holden P., Hansen U. (2013). The Expanded Collagen VI Family: New Chains and New Questions. Connect. Tissue Res..

[76] Muzzin S., Timis E., Doliana R., Mongiat M., Spessotto P. (2025). “Unraveling EMILIN-1: A Multifunctional ECM Protein with Tumor-Suppressive Roles” Mechanistic Insights into Cancer Protection Through Signaling Modulation and Lymphangiogenesis Control. Cells.

[77] Corona A., Blobe G.C. (2021). The Role of the Extracellular Matrix Protein TGFBI in Cancer. Cell. Signal..

[78] Benn M. (2009). Apolipoprotein B Levels, APOB Alleles, and Risk of Ischemic Cardiovascular Disease in the General Population, a Review. Atherosclerosis.

[79] Xu Y., Her C. (2015). Inhibition of Topoisomerase (DNA) I (TOP1): DNA Damage Repair and Anticancer Therapy. Biomolecules.

[80] Chavali S.S., Carman P.J., Shuman H., Ostap E.M., Sindelar C.V. (2025). High-Resolution Structures of Myosin-IC Reveal a Unique Actin-Binding Orientation, ADP Release Pathway, and Power Stroke Trajectory. Proc. Natl. Acad. Sci. USA.

[81] Gao Z.W., Dong K., Zhang H.Z. (2014). The Roles of CD73 in Cancer. BioMed Res. Int..

[82] De Vega S., Iwamoto T., Yamada Y. (2009). Fibulins: Multiple Roles in Matrix Structures and Tissue Functions. Cell. Mol. Life Sci..

[83] Bauzá-Martinez J., Heck A.J.R., Wu W. (2021). HLA-B and Cysteinylated Ligands Distinguish the Antigen Presentation Landscape of Extracellular Vesicles. Commun. Biol..

[84] Piñero J., Ramírez-Anguita J.M., Saüch-Pitarch J., Ronzano F., Centeno E., Sanz F., Furlong L.I. (2020). The DisGeNET Knowledge Platform for Disease Genomics: 2019 Update. Nucleic Acids Res..

[85] Carvalho A.S., Moraes M.C.S., Na C.H., Fierro-Monti I., Henriques A., Zahedi S., Bodo C., Tranfield E.M., Sousa A.L., Farinho A. (2020). Is the Proteome of Bronchoalveolar Lavage Extracellular Vesicles a Marker of Advanced Lung Cancer?. Cancers.

[86] Hurwitz S.N., Rider M.A., Bundy J.L., Liu X., Singh R.K., Meckes D.G. (2016). Proteomic Profiling of NCI-60 Extracellular Vesicles Uncovers Common Protein Cargo and Cancer Type-Specific Biomarkers. Oncotarget.

[87] Marzan A.L., Nedeva C., Mathivanan S. (2021). Extracellular Vesicles in Metabolism and Metabolic Diseases. New Frontiers: Extracellular Vesicles; Subcellular Biochemistry.

[88] Garcia N.A., Ontoria-Oviedo I., González-King H., Diez-Juan A., Sepúlveda P. (2015). Glucose Starvation in Cardiomyocytes Enhances Exosome Secretion and Promotes Angiogenesis in Endothelial Cells. PLoS ONE.

[89] Livak K.J., Schmittgen T.D. (2001). Analysis of Relative Gene Expression Data Using Real-Time Quantitative PCR and the 2^−ΔΔCT^ Method. Methods.

[90] Carvalho A.S., Baeta H., Henriques A.F.A., Ejtehadifar M., Tranfield E.M., Sousa A.L., Farinho A., Silva B.C., Cabeçadas J., Gameiro P. (2021). Proteomic Landscape of Extracellular Vesicles for Diffuse Large B-Cell Lymphoma Subtyping. Int. J. Mol. Sci..

[91] Carvalho A.S., Ribeiro H., Voabil P., Penque D., Jensen O.N., Molina H., Matthiesen R. (2014). Global Mass Spectrometry and Transcriptomics Array Based Drug Profiling Provides Novel Insight into Glucosamine Induced Endoplasmic Reticulum Stress. Mol. Cell. Proteom..

[92] Cox J., Mann M. (2008). MaxQuant Enables High Peptide Identification Rates, Individualized p.p.b.-Range Mass Accuracies and Proteome-Wide Protein Quantification. Nat. Biotechnol..

[93] Shannon P., Markiel A., Ozier O., Baliga N.S., Wang J.T., Ramage D., Amin N., Schwikowski B., Ideker T. (2003). Cytoscape: A Software Environment for Integrated Models of Biomolecular Interaction Networks. Genome Res..

[94] Deutsch E.W., Bandeira N., Sharma V., Perez-Riverol Y., Carver J.J., Kundu D.J., García-Seisdedos D., Jarnuczak A.F., Hewapathirana S., Pullman B.S. (2020). The ProteomeXchange Consortium in 2020: Enabling ‘Big Data’ Approaches in Proteomics. Nucleic Acids Res..

[95] Perez-Riverol Y., Csordas A., Bai J., Bernal-Llinares M., Hewapathirana S., Kundu D.J., Inuganti A., Griss J., Mayer G., Eisenacher M. (2019). The PRIDE Database and Related Tools and Resources in 2019: Improving Support for Quantification Data. Nucleic Acids Res..

