# Supplementary material for: Integrative Proteomics of Extracellular Vesicles from hiPSC-Derived Cardiac Organoids Reveals Heart Tissue-like Molecular Representativity
Source: Int J Mol Sci. 2026 Jan 19;27(2):981. doi: 10.3390/ijms27020981 (PMC12842532; doi:10.3390/ijms27020981)
Supplement: Supplementary file 1 [file ijms-27-00981-s001.zip › ijms-4084456-supplementary/Supplementary Material_Revised/Supplementary Figure S1.pdf]

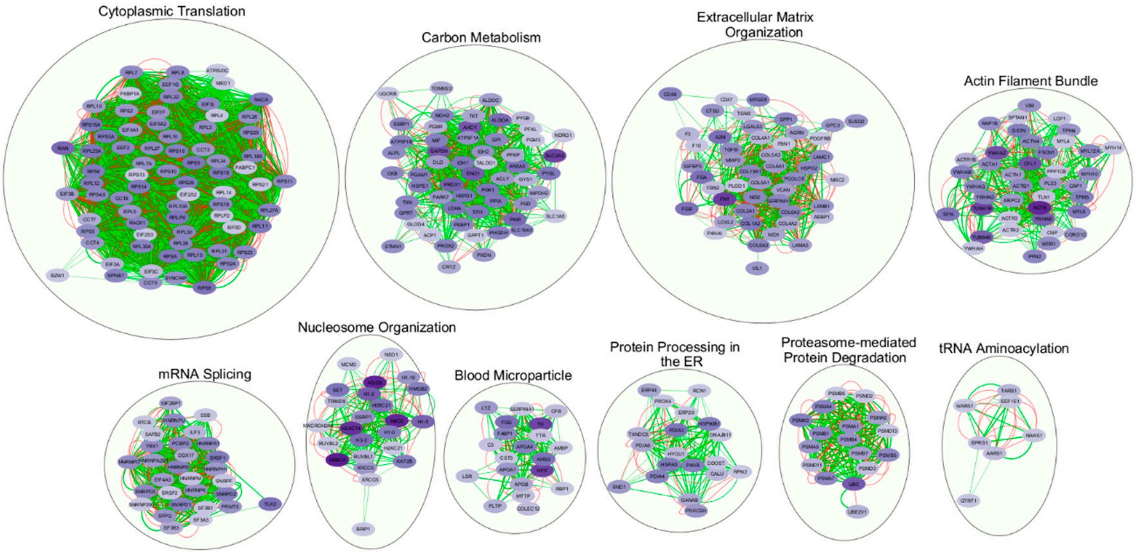

Figure S1. Ten largest groups of protein-protein interaction groups extracted from the cardEVs proteome. Proteins are represented in nodes and colored according to their Log_{10}(iBAQ) value. Green edges represent protein interactions retrieved from the STRING app, while red edges represent additional protein interactions reported on the human interactome of reference (HumanNet v3 – FN). Proteins were grouped together based on their interaction score and groups were annotated using AutoAnnotate.
