# Supplementary material for: Integrative Proteomics of Extracellular Vesicles from hiPSC-Derived Cardiac Organoids Reveals Heart Tissue-like Molecular Representativity
Source: Int J Mol Sci. 2026 Jan 19;27(2):981. doi: 10.3390/ijms27020981 (PMC12842532; doi:10.3390/ijms27020981)
Supplement: Supplementary file 1 [file ijms-27-00981-s001.zip › ijms-4084456-supplementary/Supplementary Material_Revised/Supplementary Tables S6-S9.pdf]

Table S6. Primary antibodies used for immunofluorescence.

Target	Host species	Manufacturer	Catalog number	Concentration
α-Actinin	Mouse	Sigma-Aldrich	A7811	1:500
cTnT	Mouse	Thermo Fisher	MA5-12960	1:250
VE-Cadherin	Rabbit	Abcam	AB33168	1:100
WT1	Rabbit	Cell Signaling Technology	83535	1:250
Vimentin	Goat	Abcam	AB11256	1:200

Table S7. Secondary antibodies used for immunofluorescence.

Target species	Host species	Conjugate	Manufacturer	Catalog number	Concentration
Rabbit	Donkey	Alexa Fluor 488	Invitrogen	A21206	1:250
Goat	Donkey	Alexa Fluor 488	Invitrogen	A21203	1:250
Mouse	Donkey	Alexa Fluor 594	Invitrogen	A11055	1:250

Table S8. Primary antibodies used for western blot.

Target	Host species	Manufacturer	Catalog number	Concentration
Alix	Goat	SICGEN	AB0327	1:1,100
CD63	Goat	SICGEN	AB0047	1:1,200
Syntenin-1	Rabbit	Abcam	AB133267	1:1,200

Table S9. Secondary antibodies used for western blot.

Target species	Host species	Conjugate	Manufacturer	Catalog number	Concentration
Goat	Rabbit	HRP	Sigma-Aldrich	A5420	1:10,000
Rabbit	Goat	HRP	Sigma-Aldrich	A9169	1:5,000
